# Knowledge of Acne among Medical Students: Pretest and Posttest Assessment

**DOI:** 10.1155/2014/727981

**Published:** 2014-01-28

**Authors:** Kanakapura Nanjundaswamy Shivaswamy, Arakali Lakshminarayana Shyamprasad, Tharayil Kunneth Sumathy, Chandrashekaran Ranganathan, Shanmugan Praveen Kumar

**Affiliations:** Department of Dermatology & STD, M. S. Ramaiah Medical College, Bangalore, Karnataka 560054, India

## Abstract

*Background.* Acne vulgaris is a disorder of sebaceous glands mainly affecting the adolescent population. There are some misconceptions about acne not only in the general population but also among the medical students. *Methods.* Second year medical undergraduate students attending dermatology postings for the first time were included in the study. A questionnaire (in yes or no answer format) with 20 questions on acne, each carrying one mark, was to be answered by the students. The students were categorized into 4 grades based on the marks obtained: Grade I 90% marks and above, Grade II 75%–90%, Grade III 50%–74%, and Grade IV <50% marks obtained. *Results.* Of the 144 students of the batch, 95 (69.5%) completed both pretest and posttest questionnaires. The average pretest score was 14.1 and that of the posttest was 16.9. The percentage of improvement in mean score from pretest to posttest was 16.5. Fischer's exact test was applied to analyze the improvement in scores between pretest and posttests which is significant at *P* = 0.015 (*P* < 0.05). In the paired *t*-test the improvement in mean scores between pretest and posttest was significant at *P* < 0.001.

## 1. Introduction

What is known? It is known that the knowledge of acne among general population is low. Acne vulgaris is a disorder of sebaceous glands mainly affecting the adolescent population. Up to 91% of adolescent male and 79% of adolescent females and 3% of adult male and 12% of adult females are affected [1, 2]. Its prevalence among medical students varies from 56% to 62% [3]. Even though acne vulgaris is a very common problem, there are some misconceptions about this condition not only among the general population but among medical students also [1]. Hence we undertook this study to assess the knowledge of acne among medical students and to know the impact or outcome of teaching as assessed by pretest and posttest questionnaires.

## 2. Material and Methods

Second year medical under graduate students attending dermatology postings for the first time and who could complete both pretest and posttest questionnaires were included in the study. The study period was from January 2008 to December 2008. The questionnaire consisting of 20 questions was designed by us to include almost all aspects of acne, such as incidence, aetiopathogenesis, exacerbating factors, role of hormonal factors, and treatment, including myths and facts. The questionnaire had a simple yes or no answer format carrying one mark each. The students were asked to answer all the questions independently on the first day of their dermatology posting and once again at the end of their postings. The students were categorized into 4 grades based on the marks they obtained: Grade I, 90% marks and above, Grade II, 75%–89%, Grade III, 50%–74%, and Grade IV, <50% marks obtained. Pretest assessment was done to know the knowledge of acne among students and an overall assessment was done by comparing the scores obtained during pretest and posttest to know the improvement in the knowledge of acne. The results were tabulated and analyzed using SPSS version 16. Descriptive statistics, namely, mean and standard deviation was applied to describe the data distribution. Qualitative data with respect to different grades of scoring was presented using frequency and percentages. Fischer's exact test was applied to test any association between the various scoring grades between pretest and posttest. Paired *t*-test was applied to assess any significant differences in mean scores between pretest and posttest.

## 3. Results

Of the 144 students of the batch, 95 (69.5%) completed both pretest and posttest questionnaires. In the pretest, 30% of students believed this condition to be hereditary, 54% did not know that male hormones are implicated in the pathogenesis, 82% of students did relate acne to diet, and 32% believed that excessive face wash can improve acne. The average pretest score was 14.1 (±2.3) and posttest score was 16.9 (±1.8) with a minimum score being 8 and a maximum score being 20. The percentage of improvement in mean score from pretest to posttest was 16.5 (Table 1). In pretest 8 (8.4%) were in Grade I (>90% of marks), 34 (35.8%) in Grade II (75%–89% marks), 50 (52.6%) in Grade III (50%–74% marks), and 3 (3.2%) in Grade IV (<50% marks). In the posttest 37 (38.9%) were in grade I, 47 (49.5%) were in Grade II, 11 (11.6%) were in Grade III, and none were in Grade IV (Table 2).

Of the 3 who were in Grade IV in pretest, 1 (33.3%) secured Grade I and 2 (66.7%) secured Grade II in posttest. Of the 50 who were in Grade III in pretest 14 (38%) moved to grade I, 29 (58%) to grade II and 7 (14%) remained in the same grade in posttest. Of the 34 who were in grade II in pretest, 14 (41.2%) secured grade I, 16 (47.1%) remained in the same grade, and 4 (11.8%) declined to grade III in posttest. All the 8 who were in grade I in pretest remained in the same grade in posttest with significant increase in their marks (Table 3).

Fischer's exact test was applied to analyze the improvement in scores between pretest and posttest which was significant at *P* = 0.015 (*P* < 0.05). The paired *t*-test was applied to know improvement in mean scores between pretest and posttest which was significant at *P* < 0.001.

## 4. Discussion 

Acne vulgaris is a disorder of sebaceous glands mainly affecting the adolescent population. It is estimated that up to 91% of males and 79% of females are affected among the general population. Its prevalence is about 56% to 62% amongst medical students with no significant gender predilection [1, 3]. It has been hypothesized that misconceptions prevail not only in the general population but also among medical students [4–6]. Ali et al. in their study showed that 21.7% of students believed that hereditary factors are responsible for acne as compared to 30% in our study [5]. Al Robaee noted in his study that 56% of students had adequate knowledge about acne and most believed that the factor responsible for acne was hormones and the common aggravating factor was stress as compared to 44% in our study [7]. Farid-ur-Rehman and Niazi in their study found that 79% relate acne to diet as compared to 82% in our study [1]. Eighty-two percent believed that washing face frequently would clear acne as compared to 32% in our study [8]. Our study not only points out misconceptions about acne among medical students but also shows improvement in overall knowledge about acne at the end of their clinical postings. The students' knowledge about acne was assessed using pretest and posttest questionnaires at the beginning and end of their dermatology postings. The average pretest score was 14.1 and that of posttest was 16.9. There was an increase of up to 16.5% in mean scores from pretest to posttest. This improvement was significant at *P* < 0.001 on applying paired *t*-test, indicating that there has been a significant difference in mean scores between pretest and posttest. It was also observed that students who were in lower grades in pretest improved considerably by securing higher grades in the posttest and those who were in higher grades in pretest continued to be in higher grades with significant improvement in their posttest scores as analysed using Fischer's exact test (*P* = 0.015) indicating that there is significant increase in number of students securing higher grades of marks.

What is new? The knowledge about acne among medical students is limited and improved significantly after clinical postings.

## 5. Conclusion 

This is one of the first assessment studies based on knowledge of acne among medical students. This study highlights the impact of clinical teaching which not only imparts knowledge but also eliminates misconceptions.

## Figures and Tables

**Table 1:** Descriptive statistics of pretest and posttest scores.

	*N*	Minimum	Maximum	Mean	Std. deviation
Pretest	95	8	19	14.11	2.309
Posttest	95	10	20	16.88	1.809

**Table 2:** Distribution of scoring grades between pretest and posttest.

	Pretest *N* (%)	Posttest *N* (%)
90% and above (I)	8 (8.4)	37 (38.9)
75%−89% marks (II)	34 (35.8)	47 (49.5)
50%−74% marks (III)	50 (52.6)	11 (11.6)
Less than 50% marks (IV)	3 (3.2)	0

Total	95 (100)	95 (100)

**Table 3:** Pretest and posttest grade cross tabulation.

Pretest grades		Posttest grades				Total
		90% and above (I)	75%–89% marks (II)	50%–74% marks (III)	<50% marks (IV)	
90% and above (I)	8 (8.4)	8 (100)	—	—	—	8 (100)
75%–89% (II)	34 (32.8)	14 (41.2)	16 (47.1)	4 (11.8)	—	34 (100)
50%–74% (III)	50 (50.2)	14 (38)	29 (58)	7 (14)	—	50 (100)
<50% marks (IV)	3 (3.2)	1 (33.3)	2 (66.7)	—	—	3 (100)

Total	95 (100)	37 (38.9)	47 (49.5)	11 (11.6)	—	95 (100)

## References

[1] Farid-ur-Rehman, Niazi NAK (2007). Beliefs and perceptions about acne among undergraduate medical students. *Journal of Pakistan Association of Dermatologists*.

[2] Goulden V, Stables GI, Cunliffe WJ (1999). Prevalence of facial acne in adults. *Journal of the American Academy of Dermatology*.

[3] Gonçalves G, Amado JM, Matos ME, Massa A (2012). The prevalence of acne among a group of Portuguese medical students. *Journal of the European Academy of Dermatology and Venereology*.

[4] Green J, Sinclair RD (2001). Perceptions of acne vulgaris in final year medical student written examination answers. *Australasian Journal of Dermatology*.

[5] Ali G, Mehtab K, Sheikh Z (2010). Beliefs and perceptions of acne among a sample of students from Sindh Medical College, Karachi. *Journal of the Pakistan Medical Association*.

[6] Tan JK, Vasey K, Fung KY (2001). Beliefs and perceptions of patients with acne. *Journal of the American Academy of Dermatology*.

[7] Al Robaee AA (2005). Prevalence, knowledge, beliefs and psychosocial impact of acne in University students in Central Saudi Arabia. *Saudi Medical Journal*.

[8] Smithard A, Glazebrook C, Williams HC (2001). Acne prevalence, knowledge about acne and psychological morbidity in mid-adolescence: a community-based study. *British Journal of Dermatology*.

